# Mast Cells Induce Blood Brain Barrier Damage in SCD by Causing Endoplasmic Reticulum Stress in the Endothelium

**DOI:** 10.3389/fncel.2019.00056

**Published:** 2019-02-19

**Authors:** Huy Tran, Aditya Mittal, Varun Sagi, Kathryn Luk, Aithanh Nguyen, Mihir Gupta, Julia Nguyen, Yann Lamarre, Jianxun Lei, Alonso Guedes, Kalpna Gupta

**Affiliations:** ^1^Vascular Biology Center, Division of Hematology, Oncology and Transplantation, Department of Medicine, Medical School, University of Minnesota, Minneapolis, MN, United States; ^2^Department of Neurosurgery, University of California, San Diego, San Diego, CA, United States; ^3^Anesthesia and Pain Medicine, Veterinary Clinical Science Department, College of Veterinary Medicine, University of Minnesota Twin Cities, St. Paul, MN, United States

**Keywords:** blood brain barrier, endoplasmic reticulum stress, endothelial cell, mast cell, P-selectin, sickle cell disease

## Abstract

Endothelial dysfunction underlies the pathobiology of cerebrovascular disease. Mast cells are located in close proximity to the vasculature, and vasoactive mediators released upon their activation can promote endothelial activation leading to blood brain barrier (BBB) dysfunction. We examined the mechanism of mast cell-induced endothelial activation *via* endoplasmic reticulum (ER) stress mediated P-selectin expression in a transgenic mouse model of sickle cell disease (SCD), which shows BBB dysfunction. We used mouse brain endothelial cells (mBECs) and mast cells-derived from skin of control and sickle mice to examine the mechanisms involved. Compared to control mouse mast cell conditioned medium (MCCM), mBECs incubated with sickle mouse MCCM showed increased, structural disorganization and swelling of the ER and Golgi, aggregation of ribosomes, ER stress marker proteins, accumulation of galactose-1-phosphate uridyl transferase, mitochondrial dysfunction, reactive oxygen species (ROS) production, P-selectin expression and mBEC permeability. These effects of sickle-MCCM on mBEC were inhibited by Salubrinal, a reducer of ER stress. Histamine levels in the plasma, skin releasate and in mast cells of sickle mice were higher compared to control mice. Compared to control BBB permeability was increased in sickle mice. Treatment of mice with imatinib, Salubrinal, or P-selectin blocking antibody reduced BBB permeability in sickle mice. Mast cells induce endothelial dysfunction *via* ER stress-mediated P-selectin expression. Mast cell activation contributes to ER stress mediated endothelial P-selectin expression leading to increased endothelial permeability and impairment of BBB. Targeting mast cells and/or ER stress has the potential to ameliorate endothelial dysfunction in SCD and other pathobiologies.

## Introduction

Neurovascular networks and blood brain barrier (BBB) play a critical role in cerebrovascular dysfunction underlying many pathobiological conditions including cognitive impairment and stroke (Zhang J. H. et al., 2012; Zhao et al., 2015; Raja et al., 2018). One of the genetically inherited conditions with high childhood mortality due to stroke and cognitive impairment is sickle cell disease (SCD; Gold et al., 2008). SCD is a genetic disorder arising from a substitution of valine for glutamic acid at position six on the β-globin gene (Ingram, 1956). A unique hallmark of SCD is vaso-occlusive crisis (VOC) during which sickle red blood cells (RBCs) cluster and occlude blood vessels leading to impaired oxygen supply to the organs, causing end organ damage and acute pain (Platt et al., 1994; Frenette and Atweh, 2007; Tran et al., 2017). Vascular dysfunction is a common feature of several comorbidities of SCD including lung injury, impaired BBB permeability and stroke (Hebbel et al., 2004; Manci et al., 2006; Jordan and Debaun, 2018). Endothelial activation plays a key role in the pathobiology of SCD replete with inflammation and oxidative stress (Hebbel et al., 2004). One known feature of endothelial activation in SCD is the overexpression of cell adhesion molecules (CAMs), which also contributes to VOC (Embury et al., 2004; Manwani and Frenette, 2013).

Amongst the CAMs, P-selectin expressed on endothelial cell (EC) membranes plays a critical role in microvascular blood flow and VOC in SCD (Embury et al., 2004; Kutlar and Embury, 2014). Anti-P-selectin antibody, Crizanlizumab reduced VOC in patients with SCD (Ataga et al., 2017). However, mechanisms causing endothelial P-selectin expression and endothelial activation in SCD remain poorly understood.

Mast cells have been shown to increase E- and P-selectin expression on the endothelium (Kubes and Granger, 1996). Mast cells, the tissue-resident granulocytes, release vasoactive, inflammatory, and neuromodulatory mediators including histamine, proteases, cytokines, and neuropeptides such as substance P (SP) upon activation (Aich et al., 2015). Histamine, a mediator released by mast cells, upregulates endothelial P-selectin *in vitro* and *ex vivo*, and participates in regulating P-selectin-mediated extravasation of immune cells (Jones et al., 1993). Mast cells are located in close proximity to the vasculature, and can cause endothelial activation, plasma extravasation, vasodilatation, and vascular dysfunction (Gupta and Harvima, 2018). We have previously observed that mast cell activation in HbSS-BERK mice contributes to neurogenic inflammation, resulting in increased vascular permeability (Vincent et al., 2013). HbSS-BERK mice express human α and βS globin chains with >99% human sickle hemoglobin, but no murine α or β globins (Paszty et al., 1997). Similar to patients with sickle cell anemia, HbSS-BERK mice demonstrate hemolysis, extensive organ damage, shortened life span and pain (Kohli et al., 2010; Cain et al., 2012). Increased permeability in the BBB has been observed in HbSS-BERK mice (Manci et al., 2006). Moreover, IL1β, TNFα and SP are significantly increased in transgenic humanized sickle mice and patients with SCD compared to non-sickle controls (Michaels et al., 1998; Hebbel et al., 2004; Vincent et al., 2013; Brandow et al., 2016; Campbell et al., 2016; Douglas, 2016; Wang et al., 2016; Solovey et al., 2017). Recent elegant studies have demonstrated that SP and IL33 stimulate TNFα and IL1β release from mast cells (Taracanova et al., 2017, 2018). Mast cells have been observed in brain parenchyma in some pathological conditions (Gupta and Harvima, 2018). It is therefore likely that mast cell activation may contribute to increased BBB permeability in SCD.

One of the known triggers of endothelial dysfunction, inflammation, and oxidative stress is endoplasmic reticulum (ER) stress (Lenna et al., 2014). We hypothesized that in a sickle microenvironment, mediators derived from activated mast cells contribute to endothelial dysfunction and impaired BBB by stimulating ER stress. Disturbance of the equilibrium between ER protein load and folding capacity can lead to the accumulation of misfolded proteins (Lenna et al., 2014). This accumulation of misfolded proteins activates one of the ER-stress sensors, protein kinase RNA-like ER kinase (PERK), which subsequently phosphorylates the eukaryotic translation initiation factor 2 alpha (eIF2α) and attenuates global protein synthesis. The translation of activating transcription factor-4 (ATF-4), however, is increased in response to eIF2α phosphorylation. In chronic ER stress, PERK-ATF4 pathway promotes the transcription of C/EBP homologous protein (CHOP), resulting in increased inflammatory cytokines and reactive oxygen species (ROS; Scheuner and Kaufman, 2008; Lenna et al., 2014). We therefore examined the ability of mast cells to stimulate ER-stress mediated endothelial P-selectin expression leading to impaired BBB permeability in transgenic sickle mice. We have shown earlier that mast cells are activated in the skin of sickle mice, and continue to actively degranulate following isolation *in vitro* (Vincent et al., 2013). Here, we demonstrate that mast cell activation in sickle mice stimulates P-selectin expression, increases endothelial permeability and compromises BBB permeability by inducing ER stress.

We used normal mouse brain ECs (mBEC) and transgenic BERK mice expressing either human sickle hemoglobin (called HbSS-BERK or *sickle* mice henceforth) or normal human hemoglobin A (called HbAA-BERK or *control* mice henceforth) to obtain cutaneous mast cells and examine BBB permeability.

## Materials and Methods

### Mice

Transgenic HbSS-BERK mice feature homozygous knockout of both α and β murine globins and possess transgenes for human α and β^S^ (hemoglobin S). Control HbAA-BERK mice are also knockout for both α and β murine globins but carry normal human α and β^A^ globins (hemoglobin A). Heterozygous HbAS-BERK mice are homozygous for normal human α globin, and heterozygous for human sickle β^S^ globin and human normal β^A^ globin. HbSS-BERK mice are characterized with similar pathology to human SCD, including hemolysis, reticulocytosis, anemia, extensive organ damage, reduced life span and pain (Paszty et al., 1997; Kohli et al., 2010).

It is challenging to use HbSS-BERK female mice for breeding. Therefore, HbSS-BERK male mice are mated with heterozygous HbAS females. Both sickle parents and offspring are maintained on the Sickle Diet (59M3, TestDiet, St Louis, MO, USA) up to 4–5 weeks of age and eventually changed to the regular Rodent Diet (Harlan Laboratories, Hayward, CA, USA). Litters were weaned 3 weeks after birth. Mice were housed in our AAALAC-approved, pathogen-free, climate-controlled (12 h light-to-dark cycle at 23°C) facility at the University of Minnesota. Mice were genotyped to verify the knockout of mouse globins and presence of human globins (Transnetyx, Cordova, TN, USA), and phenotyped by isoelectric focusing for the presence of HbS and/or HbA as described by us (Sagi et al., 2018). All procedures followed approved protocols from the University of Minnesota’s Institutional Animal Care and Use Committee (IACUC) and complied with the statutes of the Animal Welfare Act and the guidelines of the Public Health Service as stated in the Guide for the Care and Use of Laboratory Animals. Cannabinoid-based therapy and approaches to quantify pain in sickle cell disease; IACUC Protocol # 1306-30698A, approval date: June 24, 2013; renewed as IACUC Protocol # 1603-33542A, approval date: May 24, 2016; annual continuing review: May 10, 2018.

### Reagents

Roswell Park Memorial Institute 1640 Medium (RPMI; 72400047), Dulbecco’s Modified Eagle Medium (DMEM; 11995065), fetal bovine serum (FBS; 10438026), and cell culture supplements were from Life Technologies (Grand Island, NY). Salubrinal (SML0951), collagenase Type II (6885), hyaluronidase (H3506), protease (P8811), deoxyribonuclease I (DN25), Percoll (P1644), recombinant mouse stem cell factor (S9915) and general chemicals were obtained from Sigma-Aldrich (St. Louis, MO, USA).

### Growth and Treatment Media

Complete mast cell growth medium (RPMI with 10% FBS, 1.2 mg/mL sodium bicarbonate, 2 mM *L*-glutamine, 25 mM HEPES, and 10 ng/mL recombinant mouse stem cell factor) was used to incubate sickle and control mast cells as described (Vincent et al., 2013). After 24 h of incubation, sickle and control mast cell conditioned medium (MCCM) were collected. Complete mast cell growth medium was incubated in parallel without mast cells to obtain unconditioned medium.

### Mast Cells

As described earlier mast cells were isolated from freshly collected shaved dorsal skin (1–2 g dissected into 1-cm^3^ pieces) of sickle and control mice, washed twice with RPMI and digested with 15 ml collagenase Type II (0.2 mg/mL), hyaluronidase (0.1 mg/mL), and 0.2 mg/mL protease (0.2 mg/mL) in RPMI at 37°C for 1 h with end-over-end mixing (Vincent et al., 2013). After sedimentation by gravity, the supernatant was collected and placed on ice. The tissue pellet was suspended in 15 ml of the same enzyme solution, followed by incubation at 37°C for 30 min with mixing; then 10 ml of RPMI with 0.015 mg/ml DNase was added to the tissue/enzyme solution and the incubation continued for an additional 30 min with mixing. Again, the supernatant was collected and placed on ice; the remaining tissue was rinsed with 5 ml RPMI. The combined supernatants were passed through a 70 μm filter and concentrated by centrifugation for 10 min at 200× *g* at 4°C. The cell pellet was resuspended in 1 ml RPMI medium with 0.015 mg/ml DNase and layered on 5 ml of 70% isotonic Percoll followed by centrifugation for 20 min at 500× *g* at 4°C. Mast cells in the pellet were suspended in complete mast cell growth medium. Purity of mast cells was validated with toluidine blue and staining for c-kit (CD117, sc-1493; RRID:AB_631031, Santa Cruz Biotechnology, Santa Cruz, CA, USA) and FcεR1 (sc-68943; RRID:AB_2103020, Santa Cruz Biotechnology; Metcalfe, 2001; Vincent et al., 2013). After 5 days, mast cells were sub-cultured, and MCCM was collected after 24 h of incubation.

### Endothelial Cells

mBECs, a kind gift from Dr Robert Auerbach (University of Madison, WI, USA) were cultured in EC medium (DMEM supplemented with 10% FBS, sodium pyruvate, 0.02 mg/ml heparin, and 0.1% growth factor (EG-5, Vec Technologies, Rensselaer, NY, USA). Cells were characterized as endothelial on the basis of cobblestone morphology, uptake of acetylated LDL (BT-902, Biomedical Technologies, Inc, Stoughton, MA, USA) and the presence of VEGFR2/KDR (Clone JH121; MS-350-P0; RRID:AB_61321, Neomarkers-Thermo Fisher, Fremont, CA, USA; Gupta et al., 1997; Chen et al., 2006).

### Treatment of Endothelial Cells With Mast Cell Conditioned Medium and/or Salubrinal

Immortalized mBECs were treated with MCCM from HbSS-BERK-mast cells and HbAA-BERK-mast cells, or unconditioned medium diluted 1:1 with EC medium with 1% FBS without growth factors and incubated for 24 h, in the absence/presence of 5 μM Salubrinal (Boyce et al., 2005), an inhibitor of eIF-2α, which prevents downstream protein synthesis, and therefore lessens the burden on the ER, as applicable.

### Transmission Electron Microscopy for Endothelial Cells

Unconditioned medium or sickle or control MCCM-treated mBECs cultured on cover slips (Thermanox, Nunc 174950, Thermo Fisher, Waltham, MA, USA) was fixed in a solution of 3% paraformaldehyde, 1.5% glutaraldehyde, and 2.5% sucrose in 0.1 M sodium cacodylate buffer with 5 mM calcium chloride and 5 mM magnesium chloride (pH 7.4) for 1 h at room temperature, rinsed three times in 0.1 M sodium cacodylate buffer for 5 min each, and then placed in 1% osmium tetroxide in 0.1 M sodium cacodylate buffer overnight at 4°C. The following day cells were rinsed with ultrapure water three times for 5 min each, and post-stained in 1% aqueous uranyl acetate for 1–2 h. Subsequently, the coverslips were rinsed in ultrapure water, dehydrated in a graded series of ethanol solutions up to 100% ethanol, and embedded in Embed 812 resin (14120, Electron Microscopy Sciences, Hatfield, PA, USA). Ultrathin sections (65 nm) were stained with uranyl acetate and lead citrate, and then examined at 75 K, with a JEOL 1200EX II electron microscope (Peabody, MA, USA).

### Immunofluorescence Microscopy of Endothelial Cells

Unconditioned medium or sickle or control MCCM-treated mBECs cultured in eight well chamber slides (ibidi USA, Madison, WI, USA) were fixed with 4% paraformaldehyde, permeabilized with 0.05% Triton X-100, for 10 min, washed with PBS containing 0.01% Tween-20 (PBS-Tw) and blocked by incubation in 3% normal donkey serum/PBS-Tw. The mBECs were incubated for 1 h at room temperature with primary antibodies diluted in 3% normal donkey serum/PBS-Tw. The following antibodies were used: ER-specific rabbit anti-galactose-1-phosphate uridylyltransferase antibody (GalT, 1:100; ab178406, Abcam, Cambridge, MA, USA), Golgi-specific rabbit anti-Giantin antibody (1:100; ab24586; RRID:AB_448163, Abcam), goat anti-P-selectin (1:100; AF737; RRID:AB_2285644, R&D, Minneapolis, MN, USA). Slides were washed and then incubated for 1 h at room temperature with species-specific donkey secondary antibodies conjugated with Cy2 and Cy3 (1:200; Jackson ImmunoResearch Laboratories, West Grove, PA, USA) diluted in 3% normal donkey serum/PBS-Tw to visualize the immunoreactive proteins. Samples were mounted with Vectashield (H-1000, Vector Labs, Burlingame, CA, USA) and fluorescence images were captured using Olympus IX 70 inverted microscope (Olympus Corporation, Center Valley, PA, USA).

### Mitochondrial Membrane Potential

Unconditioned medium or sickle or control MCCM-treated mBECs were stained with 5,5′,6,6′-tetrachloro-1,1′,3,3′-tetraethylbenzimi- dazolylcarbocyanine iodide (JC-1, MitoProbe M34152, Life Technologies, Carlsbad, CA, USA) according to the manufacturer’s instructions. Measurement of red fluorescence (excitation/emission at 535 nm/595 nm) and green fluorescence (excitation/emission at 485 nm/535 nm) was performed using images captured with Olympus IX 70 inverted microscope (Olympus Corporation). Fluorescence was quantitated according to the total of fluorescence pixels divided by the total area, and analyzed with Photoshop (Adobe, San Jose, CA, USA). The ratio of red to green fluorescence was calculated and this ratio increases as mitochondrial membrane potential increases (Kimura and Murakami, 2014).

### Reactive Oxygen Species Assay

ROS formation was detected using a cell permeable fluorescent compound, 2′,7′-dichlorofluorescein diacetate (DCFDA) according to the manufacturer’s instruction (ab113851, Abcam). In brief, mBECs were seeded on a 96 well plate (clear bottom/black microplates, Nunc 165305, Thermo Fisher) and grown to 80%–85% confluence. After treatment with unconditioned medium or MCCM, the mBECs were washed twice with assay buffer and stained with 20 μM of DCFDA in assay buffer for 45 min at 37°C. Following staining with DCFDA, the cells were washed twice with PBS and the fluorescence was read immediately at 485 nm excitation and 535 nm emission on a fluorescent plate reader (Synergy HT, Biotek, Winooski, VT, USA) with Gen5™ 1.0 software (Biotek). Changes in ROS were determined as fold change in fluorescence as compared to vehicle treated control. All analyses and calibrations were performed at least in triplicate (Ye et al., 2015).

### Western Blotting

Unconditioned medium or MCCM-treated mBECs in 6-well plates were lysed with 25 mM HEPES, 300 mM NaCl, 1.5 mM MgCl, 0.2 mM EDTA, 0.1% Triton X-100, 5 mM DTT, pH 7.6 with protease inhibitors. Whole cell lysates (15–40 μg of protein) were resolved by 3%–15% SDS-PAGE and transferred to a polyvinylidene difluoride membrane (Immobilon-P, IPVH00010, Millipore, Bedford, MA, USA). The membrane was blocked and then probed with primary antibodies overnight at 4°C. The antibodies used were antibodies against phospho-eIF2α (Ser51; 9721; RRID:AB_330951), PERK (3192; RRID:AB_2095847), phospho-PERK (Thr980; 3179; RRID:AB_2095853; all from Cell Signaling Technology, Beverly, MA, USA); eIF2α (sc-11386; RRID:AB_640075), glucose regulated protein 78 (GRP78; sc-13968; RRID:AB_2119991), NADPH oxidase 4 (NOX4; sc-30141; RRID:AB_2151703), spliced X-box binding protein (sXBP1; sc-7160; RRID:AB_794171; all from Santa Cruz Biotechnology); CHOP (MA1-250; RRID:AB_2292611, Pierce-Thermo Fisher); ATF4 (ARP37017_p050; RRID:AB_593104, Aviva Systems Biology, San Diego, CA, USA), and glyceraldehyde-3-phosphate dehydrogenase (GAPDH; G9545; RRID:AB_796208, Sigma-Aldrich). After incubation with an alkaline phosphatase-conjugated donkey secondary antibody (sc-2083, Santa Cruz Biotechnology) for 60 min at room temperature the membranes were washed and the immuno-reactive proteins were detected with the ECF Western blotting system (RPN5785, GE Healthcare Life Sciences, Piscataway, NJ, USA). Chemifluorescence signals were acquired using a Storm 860 PhosphorImager (Molecular Dynamics, Sunnyvale, CA, USA). Chemifluorescence of each lane was quantified by densitometry in arbitrary units using ImageJ software (National Institutes of Health).

### Determination of Endothelial Permeability

The mBECs were cultured on 0.2% gelatin treated Transwell inserts (Corning 3384) in 96 well plates (Corning 3382). At 80%–85% confluence the cells were made quiescent by incubation with EC medium with 1% FBS and no growth factor for 18–20 h. Quiescent mBECs were incubated with 100 μl of 5 μM Salubrinal or vehicle control for 30 min in phenol red free EC medium (phenol red free DMEM, 21063029, Life Technologies) with 1% FBS in the luminal (upper) chamber. The pretreatment was replaced with 100 μl solution of unconditioned medium or MCCM diluted 1:1 with phenol red free EC medium with 1% FBS which also contained the inhibitor or vehicle and incubation was continued up to 8 h. Then, the Transwell inserts were washed briefly with PBS containing Ca^2+^/Mg^2+^. Next 100 μl Evans blue (0.6 mg/ml) bound to 0.4% BSA in PBS with Ca^2+^/Mg^2+^ was added to the upper chamber and 200 μl PBS with Ca^2+^/Mg^2+^ was added to the lower chamber. After 30 min of incubation, the absorbance of the lower chamber was determined at 620 nm with a microplate reader (Synergy HT, Biotek; Friedl et al., 2002; Garcia et al., 2011).

### Treatment of Mice With Endoplasmic Reticulum Stress Inhibitor, Salubrinal

Salubrinal was reconstituted in DMSO to make the stock solution of 20 mg/ml which was subsequently diluted in saline (0.9% NaCl) to prepare injection solution of 100 μg/ml. The mice were intraperitoneally injected with 1 mg/kg Salubrinal.

### Treatment of Mice With Imatinib

Mice were treated daily for 5 days with 100 mg/kg body weight imatinib mesylate (Gleevec, NDC 0078-0401034; Novartis) *via* gavage.

### Treatment of Mice With P-selectin Blocking Antibody

Mice were treated with 1 mg/kg body weight P-selectin blocking antibody (RB40.32, BD Biosciences) for 3 days *via* intravenous injection.

### Determination of Blood Brain Barrier Permeability

After respective treatments, mice were injected with 16.7 mg/kg FITC-Dextran 10 kDa (FD10S, Sigma-Aldrich) 1 h before euthanasia (Egawa et al., 2013). Brains were collected, homogenized in 50 mM Tris-Cl (pH = 7.6; 1 μl/mg brain) and centrifuged at 16,000 *g* for 30 min at 4°C. Fifty microliter of supernatant was transferred to a 96 well black polystyrene assay plate (Corning 3915) for analysis. A series of standards containing 0.005, 0.02, 0.1, 0.5, 2.5, 5 and 10 μg/ml FITC-Dextran 10 kDa in 50% Tris-HCl/50% absolute methanol were used. The concentration of FITC was determined by spectrofluorometry with an excitation of 485 nm (20 nm bandwidth) and an emission wavelength of 528 nm (20 nm bandwidth).

### Histamine Analysis

Skin punch biopsies (4 mm) were collected from the dorsal skin of mice immediately after euthanasia. Biopsies were incubated in DMEM plus antibiotics with 2 mM *L*-glutamine and 10 mM HEPES (Thermo Fisher) for 24 h at 37°C in a 5% CO_2_ incubator. The conditioned media was snap-frozen and stored at −80°C until analyzed.

Whole blood was collected by cardiac puncture into EDTA tubes (T-MQK, Terumo Medical Corp., Somerset, NJ, USA). Plasma was separated by centrifugation of whole blood at 1,200 *g* and 4°C for 10 min. Equal volumes of plasma and methanol were added to a 1.5 ml microcentrifuge tube, mixed, and incubated on ice for 3 min. Supernatant was collected after the mixture was centrifuged at 5,000 *g* for 5 min. The extract was then dried in a centrifugal concentrator for 2–3 h at room temperature and reconstituted with assay buffer prior to analysis.

The processed sample was analyzed using the Histamine ELISA kit (ENZ-KIT140-0001, Enzo LifeScience). Assay results were collected and calculated using the Synergy HT plate reader and Gen5™ 1.0 data analysis software (BioTek).

### Statistical Analysis

All data were analyzed using Prism software (v 6.0a, GraphPad Software Inc., San Diego, CA, USA). A two-way repeated measures analysis of variance (ANOVA) with Bonferroni’s correction was used to compare the responses among treatments. A *p*-value of < 0.05 was considered significant. All data are presented as mean ± SEM.

## Results

### Mast Cells From Sickle Mice Induce Endoplasmic Reticulum Stress in Endothelial Cells

**Figure 1:**
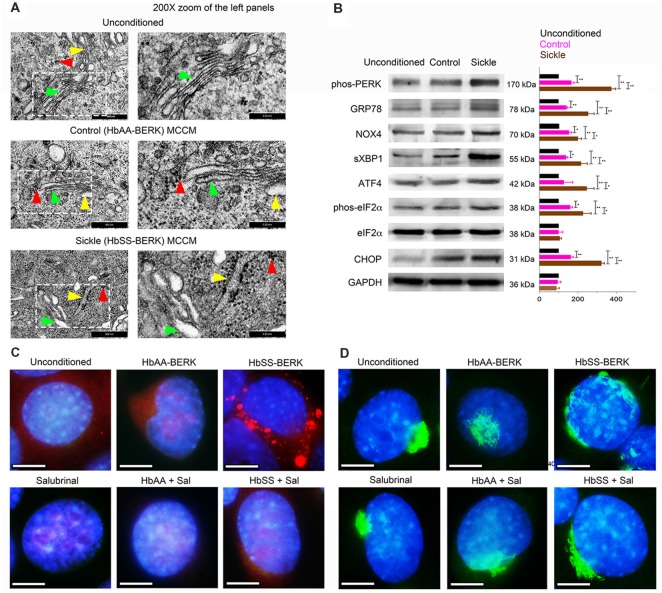
Mast cells from sickle mice induce endoplasmic reticulum (ER) stress in endothelial cells (ECs). Mouse brain ECs (mBECs) were treated for 24 h with mast cell conditioned medium (MCCM) from cultures of mast cells isolated from the skin of sickle or control mice. mBECs were pre-treated with Salubrinal (5 μM) or vehicle for 30 min before addition of the MCCM, where applicable. **(A)** Transmission electron microscopic images of ER (yellow arrows), Golgi (green arrows), and ribosomes (red arrows) in mBECs. Left column, Magnification ×75,000; scale bar = 500 nm. Right column shows 200× magnification of the inset in the left column; scale bar = 0.25 nm. Each image represents five separate and reproducible experiments. **(B)** Sickle and control MCCM stimulate an increase in ER stress markers. Western blot analysis of phos-protein kinase-R-like ER kinase (PERK), glucose regulated protein-78 (GRP-78), NADPH oxidase 4 (NOX4), spliced X-box binding protein (sXBP1), activating transcription factor 4 (ATF4), phos-eukaryotic translation initiation factor 2 alpha (eIF2α), eIF2α, C/EBP homologous protein (CHOP) and protein-loading control GAPDH. Each band is a representative of three independent and reproducible experiments. Each bar is the mean ± SEM of percentage of band density of indicated protein to that of GAPDH from three separate experiments. *p < 0.05; **p < 0.01. **(C)** Sickle MCCM-induced accumulation of proteins in mBEC is abrogated by Salubrinal. mBEC stained for the ER marker, Galactose-1-phosphate uridylyltransferase (GalT; red) and nuclei (DAPI, blue). Each image represents images from five separate and reproducible experiments. Scale bar = 10 μm; magnification 150×. **(D)** Loss of Golgi organization is attenuated by Salubrinal in mBEC treated with sickle MCCM. Representative images of Golgi (Giantin; green) and nuclei (DAPI, blue). Magnification 150× and scale bar = 10 μm. Each image represents reproducible images from five separate experiments. Sal, Salubrinal.

Normal mBECs were incubated with MCCM from mast cell cultures derived from sickle and control mice. In parallel, mBECs were also incubated with unconditioned mast cell culture medium incubated without mast cells. Transmission electron microscopy of mBECs incubated with unconditioned medium showed the normal presentation of the ER (yellow arrowheads) and Golgi (green arrowheads) with well-organized stacks of cisternae and few sparsely scattered ribosomes (red arrowheads; Figure 1A). MCCM from control mice induced partial disruption and swelling of ER and Golgi cisternae with prominent dark granular ribosomes (Figure 1A). mBECs treated with sickle-MCCM exhibited pronounced swelling and structural disorganization of Golgi and ER, accompanied by aggregation of dense clusters of ribosomes around the ER and throughout the cytoplasm (Figure 1A). Extensive ribosomal aggregates indicate accumulation of misfolded proteins due to malfunctioning of the ER and Golgi (Hiramatsu et al., 2015; Oakes and Papa, 2015). Therefore, these observations suggest that mast cell released mediators stimulate ER stress in mBECs which was higher with MCCM from sickle mouse mast cells compared to that of control mice.

Complementary to transmission electron microscopy, Western blotting demonstrated significantly enhanced expression of ER stress markers, phos-PERK, GRP78, NOX4, sXBP1, ATF4, phos-eIF2α, and CHOP in mBECs incubated with sickle MCCM, compared to those incubated in control MCCM or unconditioned MCCM (Figure 1B). We also observed accumulation of galactose-1-phosphate uridylyltransferase (GalT; red) in mBECs treated with sickle MCCM, but not in those treated with control MCCM or unconditioned medium (Figure 1C). GalT accumulation in the ER, instead of being secreted and distributed throughout the cell, is indicative of accumulation of unfolded proteins under ER stress. Similarly, complete loss of structural integrity of Golgi (Giantin; green) was observed in mBECs treated with sickle MCCM, but not in those treated with control MCCM or unconditioned medium (Figure 1D). Salubrinal has been shown to increase levels of phos-eIF2α, which prevents downstream protein synthesis, by blocking dephosphorylation after ER stress induced phosphorylation (Boyce et al., 2005; Lewerenz and Maher, 2009). We performed a functional assay to examine the impact of Salubrinal on unfolded protein accumulation. Salubrinal abrogated the accumulation of GalT and reduced the changes in Golgi (Figures 1C,D). Therefore, sickle MCCM contributes to ER stress in mBECs by inducing structural changes in the ER-Golgi complex *via* functionally activating unfolded protein response pathways. These processes are attenuated by reducing ER stress with Salubrinal.

### Mast Cells Contribute to Endothelial Mitochondrial Dysfunction and Oxidative Stress

**Figure 2:**
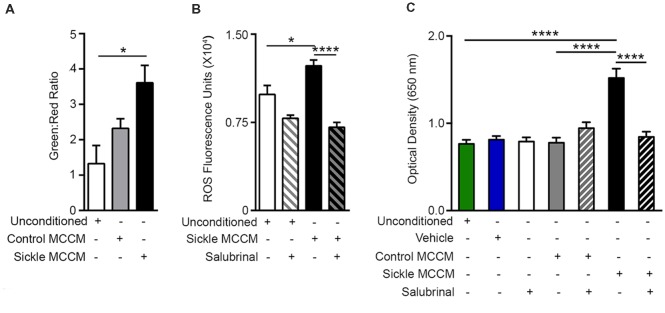
Mast cell induced endothelial ER stress is accompanied by mitochondrial dysfunction, reactive oxygen species (ROS) production, and increased permeability. mBECs were treated for 24 h with MCCM from cultures of mast cells isolated from the skin of sickle or control mice. mBECs were pre-treated with Salubrinal (5 μM) or vehicle for 30 min before addition of MCCM, where applicable. **(A)** Sickle MCCM significantly decreased mitochondrial membrane potential detected by JC-1. An increased green to red fluorescence ratio is indicative of decreased membrane potential. *p < 0.05. **(B)** Sickle MCCM significantly increases the production of ROS, which is ameliorated by pre-treatment with Salubrinal. ROS in mBEC is shown as fluorescence units of oxidized 2′,7′-dichlorodihydrofluorescein. **(C)** Sickle MCCM significantly increases the endothelial permeability, which is ameliorated by pre-treatment with Salubrinal. Evans blue leakage through mBEC monolayer following incubation with control or sickle MCCM, measured at 650 nm is shown. *p < 0.05, ****p < 0.0001.

ER stress is also accompanied by increased oxidative stress due to mitochondrial dysfunction and dysregulated antioxidant homeostasis (Lenna et al., 2014). Correspondingly, we observed decreased mitochondrial potential (Figure 2A; *p* = 0.0347) and increased ROS (Figure 2B; *p* = 0.0135) in mBECs treated with sickle MCCM as compared to those treated with unconditioned medium. Salubrinal inhibited ROS production in mBECs induced by sickle MCCM (Figure 2B; *p* < 0.0001). This result supports previous findings where Salubrinal promotes homeostasis by decreasing ROS production, ER stress, and mitochondrial dysfunction (Dou et al., 2012; Wu et al., 2012; Zhu et al., 2012). Salubrinal acts by preventing dephosphorylation of eIF2α and sustaining PERK-ATF4 signaling during ER stress (Boyce et al., 2005; Tsaytler et al., 2011). The mBEC monolayer treated with sickle MCCM *in vitro* demonstrated increased Evans blue leakage relative to those treated with control MCCM (*p* < 0.0001) or unconditioned medium (*p* < 0.0001), indicative of increased endothelial permeability (Figure 2C), likely caused by the action of vasoactive substances such as SP and/or histamine or other substances released from mast cells.

### Mast Cell Activation Contributes to Increased Histamine in Sickle Mice

**Figure 3:**
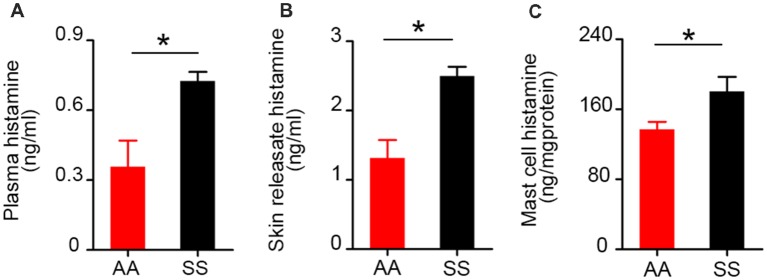
Histamine levels in plasma, skin secretagogue, and mast cells of sickle mice are higher than control mice. Histamine concentration in **(A)** plasma (*n* = 6). **(B)** Skin releasate/secretagogue (*n* = 3). **(C)** Cutaneous mast cells in culture (*n* = 6). *p < 0.05. All specimens were from ~3.5-month-old female mice. AA, HbAA-BERK control mice and SS, HbSS-BERK sickle mice.

Histamine is one of the potent inflammatory mediators released from mast cells. Mast cell histamine release is considered to be one of the major cellular mechanisms underlying histamine-induced barrier dysfunction (Kumar et al., 2009). Besides systemic inflammation, histamine is known for its critical role in neurogenic inflammation and transmission of pain throughout the nervous system (Rosa and Fantozzi, 2013). We compared control and sickle plasma, skin secretagogue, and mast cell histamine levels. We found a significant increase in histamine levels in sickle mice in the plasma (Figure 3A; *p* = 0.0119), skin secretagogue (Figure 3B; *p* = 0.0156) and mast cells (Figure 3C; *p* = 0.0368) isolated from the skin when compared to control mice.

### Sickle Mast Cell Mediators Induce P-selectin Expression on Endothelial Cells

**Figure 4:**
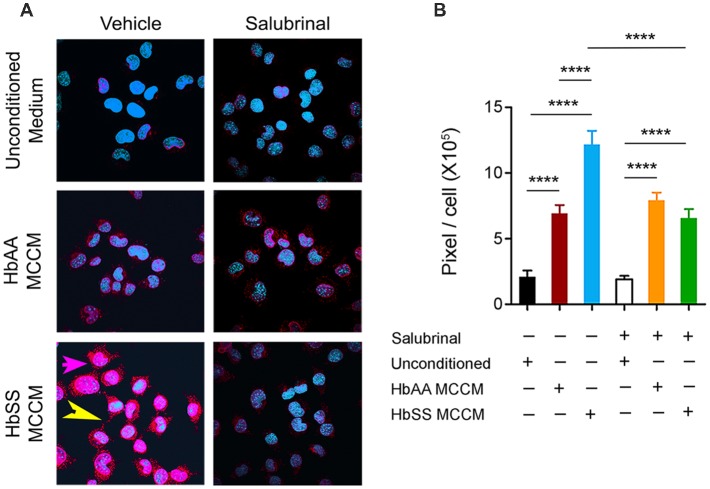
Mast cells induce P-selectin expression in ECs. mBECs were treated for 24 h with MCCM from cultures of mast cells isolated from the skin of sickle or control mice. **(A)** Representative images of P-selectin in mBECs, magnification 60× showing blue nuclei and red P-selectin staining. In bottom left panel for mBEC incubated with sickle-MCCM please note magenta-pink staining (pink arrow), indicating cell surface expression covering the entire cell surface and also red extracellular P-selectin granules (yellow arrow; **B**). Quantification of P-selectin expression on mBEC. mBECs treated with unconditioned medium, *n* = 14, black; mBECs treated with HbAA MCCM, *n* = 12, maroon; mBECs treated with HbSS MCCM, *n* = 16, blue; mBECs pre-treated with Salubrinal followed by treatment with unconditioned medium, *n* = 15, white; mBECs pre-treated with Salubrinal followed by treatment with HbAA MCCM, *n* = 12, orange; mBECs pre-treated with Salubrinal followed by treatment with HbSS MCCM, *n* = 11, green. ****p < 0.0001. HbAA, HbAA-BERK control and HbSS, HbSS-BERK sickle mice.

P-selectin is known to be upregulated by mast cell activation (Torres et al., 2002), and contributes significantly to the recruitment and rolling of leukocytes and neutrophils (Jones et al., 1993; Mayadas et al., 1993), and participates in the attachment of sickle RBCs to the endothelium (Matsui et al., 2001). mBECs incubated with control and sickle MCCM exhibited about 3- and 6-fold fold increase, respectively, in P-selectin expression compared to unconditioned culture medium (Figures 4A,B; *p* < 0.0001 and *p* < 0.0001, respectively). P-selectin expression induced by sickle-MCCM appeared to be associated with cell membrane as well as dense intracellular granules (Figure 4A). In contrast P-selectin expression induced by control-MCCM appeared to be confined as dense red intracellular staining. Pre-incubation of mBECs with Salubrinal significantly inhibited sickle MCCM-induced P-selectin expression on the cell membrane of mBECs and intracellularly (Figure 4B; *p* < 0.0001) to the level induced by control MCCM. In contrast, Salubrinal did not inhibit control MCCM-induced P-selectin expression on mBECs. It is therefore likely that mast cells from sickle mice release substances that promote translocation of P-selectin to endothelial surface which is mediated by ER stress.

### Blood Brain Barrier Permeability in Sickle Mice Is Attenuated by Reducing Endoplasmic Reticulum Stress or Blockade of P-selectin or Inhibiting Mast Cells

**Figure 5:**
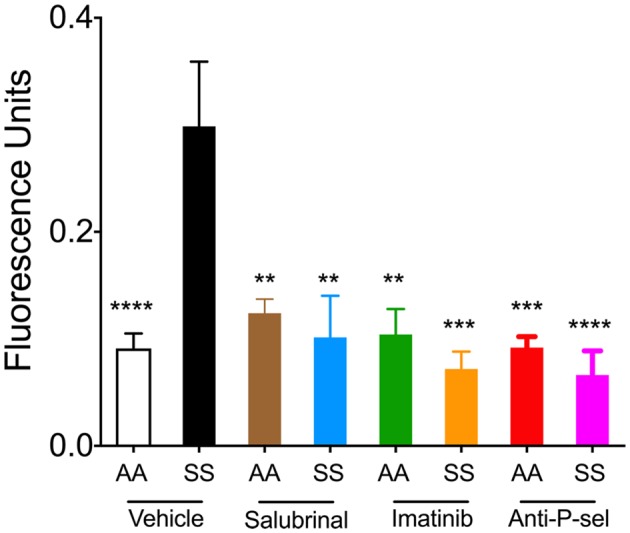
Salubrinal, imatinib, and P-selectin blocking antibody reduce blood brain barrier (BBB) permeability in sickle mice. Mice were treated with either a single dose of 1 mg/kg body weight Salubrinal for 48 h, 100 mg/kg body weight imatinib for 5 days, or 1 mg/kg body weight P-selectin blocking antibody for 3 days. After each of the respective treatments, mice were injected with 16.7 mg/kg FITC-Dextran 10 kDa through the tail vein 1 h prior to euthanasia at the end of the study. FITC-dextran leakage in the brain is shown. Control mice treated with vehicle, *n* = 8, white; sickle mice treated with vehicle, *n* = 6, black; control mice treated with Salubrinal, *n* = 4, brown; sickle mice treated with Salubrinal, *n* = 6, blue; control mice treated with imatinib, *n* = 4, green; sickle mice treated with imatinib, *n* = 3, orange; control mice treated with P-selectin blocking antibody, *n* = 4, red; sickle mice treated with P-selectin blocking antibody, *n* = 6, purple. **p < 0.01, ***p < 0.001, ****p < 0.0001 compared to sickle mice treated with vehicle. Female sickle (SS) or control (AA) mice at ~3.5 months of age were used.

We next examined the effects of Salubrinal (to reduce ER stress), imatinib (to inhibit mast cell activation), and P-selectin blocking antibody on BBB permeability in mice by examining the leakage of FITC-dextran. Sickle mice showed significantly increased extravasation of FITC-dextran compared to control mice (Figure 5; *p* < 0.0001). After 48 h of treatment with Salubrinal, a reduction in BBB permeability was observed in sickle mice when compared to the vehicle group (Figure 5; *p* = 0.017). A similar decrease was observed in sickle mice treated with imatinib for 5 days (Figure 5; *p* = 0.0008) and P-selectin blocking antibody for 3 days (Figure 5; *p* < 0.0001).

## Discussion

**Figure 6:**
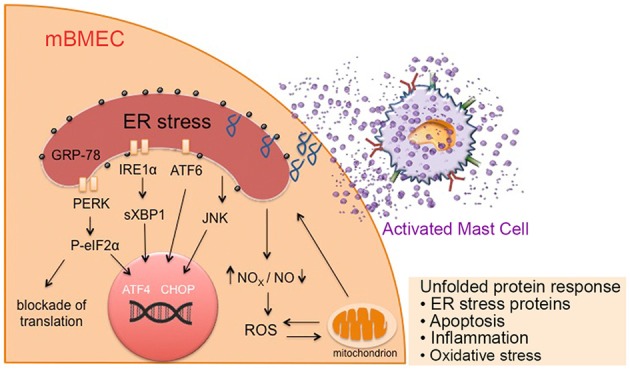
Mast cell activation in sickle cell disease (SCD) leads to ER stress, mitochondrial dysfunction, and associated oxidative stress, leading to increased endothelial permeability. Mediators released from activated mast cells in sickle mice stimulate ER stress in endothelium. These factors may directly act on the ER or increase ROS production and mitochondrial dysfunction leading to the activation of chaperone proteins involved in ER homeostasis maintenance, resulting in the unfolded protein response and accumulation of unfolded proteins in the ER. This further augments mitochondrial dysfunction and subsequent oxidative stress, resulting in inflammation, leading to a vicious cycle of inflammation, oxidative stress and mast cell activation. Prolonged ER stress may result in apoptosis. ATF6, activating transcription factor 6; CHOP, C/EBP homologous protein; GRP-78, glucose regulated protein-78; IRE1, inositol-requiring enzyme 1; mBECs, mouse brain microvascular endothelial cells; NO, nitric oxide; NOX, nicotinamide adenine dinucleotide phosphate oxidase; P-eIF2α; phosphorylated eukaryotic initiation factor 2α; PERK, protein kinase-R-like ER kinase; ROS, reactive oxygen species; sXBP1, spliced X-box binding protein.

Our data demonstrate that mast cells contribute to upregulation of endothelial P-selectin expression *via* an ER stress mediated mechanism, which leads to increased endothelial permeability and impairment of the BBB in sickle mice. For the first time we show the novel role of mast cells on endothelial activation which could have implications in multiple consequences of SCD including VOC and stroke. Mast cells are constitutively activated in sickle mice (Vincent et al., 2013). Our observations therefore evince the significance and the pivotal role of mast cells in SCD pathobiology (Figure 6).

P-selectin is a CAM known to initiate and mediate the binding of leukocytes to anchor and roll on vessel walls (Mcever et al., 1995). In sickle mice, EC surface P-selectin facilitates the adhesion of sickle RBCs to the vessel wall leading to vascular occlusion (Embury et al., 2004). Absence and/or blockade of cell surface P-selectin leads to reduced adhesion of leukocytes and sickle RBCs to the endothelium, and has an inhibitory effect on vaso-occlusion in sickle mice (Turhan et al., 2002; Embury et al., 2004; Gutsaeva et al., 2011). We noted that ECs express and maintain strong P-selectin expression on the cell surface of mBECs when incubated with MCCM from mast cells derived from sickle mice but not from MCCM from control mice mast cells. Histamine, which is a prominent mast-cell derived mediator, has been demonstrated to stimulate P-selectin expression on the endothelium, which is accompanied by increased adhesion of neutrophils to vessel walls (Sun et al., 2012). Histamine has also been shown to rapidly disrupt cell-cell and cell-extracellular matrix interaction, which affects the stability of endothelium-basal connective tissue (Moy et al., 2000). Plasma, skin releasate, and mast cell histamine levels were significantly higher in sickle mice when compared to control mice. Therefore, in SCD, histamine and other mast cell-derived mediators lead to functional overexpression of P-selectin on ECs, exaggerating the immune response, leading to inflammation and a more adherent lumen. ROS have been shown to upregulate adhesion molecules by increasing the transport of molecules such as P-selectin to the cell surface and the circulation (Lum and Roebuck, 2001). High mobility group 1B protein (HMGB1) has also been implicated in increasing the expression of P-selectin *via* ER stress (Luo et al., 2013). HMGB1 has been known to activate Toll-like receptor 4, inducing organ injury and pain in SCD (Xu et al., 2014). Mast cells have been shown to release HMGB1 after injury (Cai et al., 2011). Therefore, mast cells and their mediators increase production of P-selectins by increasing ROS and provoking ER stress as observed in this study. Thus, mast cells play a cardinal role in functional endothelial P-selectin expression in SCD. Mast cells release several mediators including cytokines, proteases, and neuropeptides such as SP upon activation (Vincent et al., 2013; Aich et al., 2015). Thus, it may be challenging to target each mediator individually, and an upstream approach inhibiting the activity of mast cells may be more appropriate. Inhibitors of mast cells as well as P-selectin have been tested clinically leading to reduced VOC in SCD without known adverse events (Kutlar et al., 2012; Kutlar and Embury, 2014; Ataga et al., 2017).

ER stress contributes to both vascular and neural pathobiology of SCD. ER stress is activated by p38 mitogen-activated protein kinases (p38MAPK) and may even suppress endothelial nitric oxide synthase, thus depleting nitric oxide (Galan et al., 2014; Santos et al., 2014). Reduced nitric oxide bioavailability is a critical feature of sickle pathobiology (Rees and Gibson, 2012). Our laboratory observed increased p38MAPK phosphorylation in the whole tissue lysates of spinal cords of HbSS-BERK mice correlative to central sensitization of spinal dorsal horn neurons, which contribute to chronic pain (Cataldo et al., 2015). Elegant studies on diabetic mice show the contribution of ER stress to neuropathic pain and inhibition with soluble epoxide hydrolase inhibitors (Inceoglu et al., 2015). ER stress contributes to hypoxia/reperfusion-induced brain damage in growing rats, which is ameliorated by Salubrinal (Cai et al., 2014). Mast cell-mediated ER stress leading to endothelial dysfunction, observed herein, may also underlie many vascular dysfunction associated complications including acute lung injury and stroke—critical co-morbidities in SCD. Mast cell activation has been shown to contribute to stroke (Arac et al., 2014) and acute lung injury in preclinical studies (Zhao et al., 2014). Silent infarcts and overt strokes are common in children with SCD (Gold et al., 2008; Rees et al., 2010). However, the mechanistic understanding of cerebral vascular dysfunction in SCD remains an enigma (Hillery and Panepinto, 2004). Our results demonstrate a fundamental mechanism of mast cell-orchestrated endothelial dysfunction *via* ER stress. Therefore, ER stress may represent a therapeutic target to ameliorate vascular dysfunction using novel pharmacologics such as Salubrinal, in addition to mast cell stabilization. Salubrinal may also have beneficial off-target effects because it has been shown to promote bone healing in rat femurs (Zhang P. et al., 2012) and increase fetal hemoglobin expression in primary human erythroid cells (Hahn and Lowrey, 2014).

Both internal and external ROS contribute to loss of EC—cell interactions (van Wetering et al., 2002), altered BBB integrity and disruption of tight junctions (Schreibelt et al., 2007; Lehner et al., 2011). In sickle mice, ROS is increased in the spinal cords and underlies the ischemia reperfusion injury (Osarogiagbon et al., 2000; Valverde et al., 2016). BBB permeability is also compromised in sickle mice (Manci et al., 2006). Our observation of increased ROS in ECs treated with sickle mast cell secretagogue and mast cell activation in sickle mice (Vincent et al., 2013) suggests that mast cell activation could compromise the BBB and allow further entry of inflammatory substances into the brain. Our findings of increased BBB permeability and mast cell-induced ER stress provide another perspective of SCD pathobiology and therapy.

In conclusion, these observations on the involvement of mast cell-induced endothelial ER stress have wide-ranging translational potential in developing therapies to co-treat organ damage and pain in SCD and cerebrovascular dysfunction in other conditions.

## Author Contributions

HT performed experiments and wrote the manuscript. AM performed experiments, analyzed and interpreted the data, and prepared figures. VS wrote the manuscript, analyzed and interpreted the data, and prepared figures. KL, AN, JN, YL and JL performed experiments. MG developed experimental plan, analyzed the data and edited the manuscript. AG advised on ER stress, interpreted the data and edited the manuscript. KG conceived, designed, planned, and supervised the entire study, analyzed and interpreted data, and edited the manuscript.

## Conflict of Interest Statement

KG is a Consultant for Tau Tona Group, Fera, Glycomimetics and Novartis but it does not conflict with the present work. The remaining authors declare that the research was conducted in the absence of any commercial or financial relationships that could be construed as a potential conflict of interest.

## Abbreviations

ATF4: activating transcription factor 4
BBB: blood brain barrier
CHOP: C/EBP homologous protein
ER: endoplasmic reticulum
GalT: galactose-1-phosphate uridylyltransferase
GRP78: glucose regulated protein 78
HMGB1: high mobility group 1B protein
mBECs: mouse brain endothelial cells
MCCM: mast cell conditioned medium
NOX4: NADPH oxidase 4
phos-eIF2α: phosphorylated eukaryotic initiation factor 2α
phos-PERK: phosphorylated protein kinase-R-like ER kinase
ROS: reactive oxygen species
SCD: sickle cell disease
SP: substance P
sXBP1: spliced X-box binding protein
VOC: vaso-occlusive crisis.
